# Epigenotoxic Effect of Dimethyl Sulfoxide on Buffalo Somatic Cells and Buffalo-Bovine Interspecies Somatic Cell Nuclear Transfer Embryos

**DOI:** 10.22074/cellj.2019.5446

**Published:** 2018-08-07

**Authors:** Husamaldeen Alsalim, Farnoosh Jafarpour, Faezeh Ghazvini Zadegan, Mohammad Hossein Nasr-Esfahani, Amir Niasari-Naslaji

**Affiliations:** 1 Department of Theriogenology, Faculty of Veterinary Medicine, University of Tehran, Tehran, Iran; 2 Department of Theriogenology, Faculty of Veterinary Medicine, University of Basra, Basra, Iraq; 3 Department of Reproductive Biotechnology, Reproductive Biomedicine Research Center, Royan Institute for Biotechnology, ACECR, Isfahan, Iran

**Keywords:** Cloning, Dimethyl Sulfoxide, DNA Methylation, Embryo, Epigenetic

## Abstract

**Objective:**

In the present study, we investigated the possible epigenotoxic effect of dimethyl sulfoxide (DMSO) on buffalo fibroblast cells and on reconstructed oocytes during buffalo-bovine interspecies somatic cell nuclear transfer (iSCNT) procedure and its effect on rate and quality of blastocyst which derived from these reconstructed oocytes.

**Materials and Methods:**

In this experimental study, cell viability of buffalo fibroblasts was assessed after exposure to various concentration (0.5, 1, 2 and 4%) of DMSO using MTS assay. The epigenetic effect of DMSO was also assessed in terms of DNA methylation in treated cells by flowcytometry. Reconstructed oocytes of buffalo-bovine iSCNT exposed for 16 hours after activation to non-toxic concentration of DMSO (0.5%) to investigate the respective level of 5-methylcytosine, cleavage and blastocyst rates and gene expression (pluripotent genes: *OCT4, NANOG, SOX2,* and trophectodermal genes: *CDX2* and *TEAD4*) of produced blastocysts.

**Results:**

Supplementation of culture medium with 4% DMSO had substantial adverse effect on the cell viability after 24 hours. DMSO, at 2% concentration, affected cell viability after 48 hours and increased DNA methylation and mRNA expression of *DNMT3A* in fibroblast cells. Exposure of reconstructed oocytes to 0.5% DMSO for 16 hours post activation did not have significant effect on DNA methylation, nor on the developmental competency of reconstructed oocyte, however, it decreased the mRNA expression of *NANOG* in iSCNT blastocysts.

**Conclusion:**

Depending on the dose, DMSO might have epigenotoxic effect on buffalo fibroblast cells and reconstructed oocytes and perturb the mRNA expression of *NANOG* in iSCNT blastocysts.

## Introduction

Embryonic development and differentiation processes in mammalians are precisely controlled by epigenetic mechanisms such as histone modifications and DNA methylation (1-3). Epigenetic reprogramming has a crucial role during embryonic and fetal development in mammals (2, 3). Any perturbation in epigenetic modifications during early and late development has negative consequences on offspring survival and health.

Dimethyl sulfoxide (DMSO) is an organosulfur and amphipathic compound that has various applications in biomedical sciences. DMSO is used widely as a solvent, for water-insoluble compounds, (4) and cryoprotectant (5). It is also used to arrest human lymphoid cells at G1 phase of cell cycle in a reversible manner (6, 7). Furthermore treatment of P19 embryonic carcinoma cells with DMSO can differentiate them into cardiomyocytes and skeletal muscle cells (8). In addition, a significant improvement in terms of blastocyst formation and full term development was observed in mouse somatic cell nuclear transfer (SCNT), following addition of 1% DMSO, as a cytokinesis inhibitor, to the activation medium of reconstructed oocytes (9). 

DMSO can regulate epigenetic mechanisms and alter CpG methylation patterns in various cells and tissues (1015). It was proposed that any remnant of DMSO in embryo preservation media may affect the epigenetic status of cells, oocytes and embryos (15-18). Supplementation of culture medium with DMSO increased an expression of mRNA and DNA methyl transferase 3A (*DNMT3A*) in embryonic bodies. It also induced hypermethylation as well as hypomethylation on genomic loci of embryonic bodies (10). Exposure of MC3T3-E1 cells for 24 hours to DMSO, increased the mRNA expression of Tet family which are responsible for hydroxylation of DNA methylation and also decreased the mRNA expression of Dnmt family which are responsible for DNA methylation (2). MII oocytes exhibited lower DNA methylation when treated with DMSO compared to glycerol (15). Activity of DNMT3A could be stimulated by the addition of DMSO. Although further enzymatic analysis suggested that the DMSO stimulation effect may depend on the interaction between DMSO and the reaction substrates (DNA and AdoMet) and not on the enzyme itself (19). 

With regard to aforementioned literature and the presumptive effect of DMSO on epigenetic characteristics of treated somatic cells and embryos, we designed this study to investigate the epigenetic effect of non-toxic dose of DMSO on buffalo fibroblast cells and reconstructed oocytes of buffalo-bovine interspecies SCNT (iSCNT) as well as the quality and rate of blastocyst derived from these reconstructed oocytes. 

## Materials and Methods

In this experimental study, unless otherwise specified, all media and chemicals were obtained from Gibco (Invitrogen Corporation, Grand Island, NY, USA) and Sigma Aldrich Chemicals (St. Louis, MO, USA), respectively. This study received an approval from Ethical Committee of Royan Institute (www.royaninstitute.org).

### Somatic donor cell preparation 

Somatic donor cells from buffalo were prepared as described previously (20). Briefly, a skin biopsy was taken from a 3-month-old female buffalo. The biopsy was cut into very tiny pieces (1-2 mm^2^) and cultured as an explant in Dulbecco’s modified Eagle medium F-12 (DMEM/F-12, Gibco, USA) with 10% fetal bovine serum (FBS, Gibco, USA) and antibiotic (1% penicillin- streptomycin) at 37°C under a humidified atmosphere of 5% CO_2_ until 80% confluency. Fibroblast outgrowths were passaged and stored in liquid nitrogen as described previously (21). For iSCNT, frozen fibroblasts were thawed and cultured in DMEM/F-12 plus 10% FBS. Synchronization of donor cells in G0 were achieved by culture in DMEM/F-12 supplemented with 0.5% FBS for 3 days. Cells from passage 2-3 were used for iSCNT experiments.

### Cytotoxicity assessment

Toxicity of different concentrations of DMSO on fibroblast cells were determined using 3-(4, 5-dimethylthiazol-2-yl)-5-(3-carboxymethoxyphenyl)2-(4-sulfophenyl)-2H-tetrazolium (MTS) assay. In brief, 5000 buffalo cells were cultured in DMEM/F-12 supplemented with 10% FBS in 96 well dish. After 24 hours, DMEM/F-12+10% FBS containing varying concentrations of DMSO (0, 0.5, 1, 2 and 4%) were added to cultured cells and incubated for 24, 48 and 72 hours. Then MTS was added to each well and incubated for 4 hours at 37°C. Absorbance ratio of various concentrations of DMSO relative to control was measured at 492 nm by using multi-well spectrophotometer. All analyses were measured in three independent replication and each replication consisted of triplicate samples.

### Semi-quantitative assessment of global DNA methylation 

The respective effects of nontoxic doses of DMSO on global DNA methylation levels of buffalo treated cells were assessed using flow cytometry through measuring fluorescence intensity of the complexes between DNAand primary and secondary antibodies in cells, as described previously (21). In brief, after treating fibroblast cells with various concentration of DMSO for 24 hours, cells were fixed with cold (4°C) 70% ethanol for 1 hour in refrigerator. Permeabilization was done using 1% Triton X-100 in phosphate buffer solution without calcium and magnesium (PBS-Gibco, USA) for 30 minutes at room temperature (RT). The cells were then treated with 4 N HCl (Sigma, USA) for 30 minutes at RT to denature the DNA. HCl was neutralized with incubation of cells with 100 mM Tris-HCl buffer (pH=8.0) for 20 minutes. In order to block non-specific binding sites, the cells were incubated in blocking solution (PBS-supplemented with 1% bovine serum albumin and 10% goat serum) for 2 hours at RT. Subsequently, cells were incubated with mouse anti-5-methyl cytosine (BI-MECY-0100, Eurogentec, Belgium, 1:400 dilution) antibodies overnight in 4°C for assessment of DNA methylation. After extensive washing, cells were incubated with goat anti-mouse IgG-fluorescein conjugated (1:50 dilution, Chemicon, AP124F) as a secondary antibody for 1 hour at 37°C. Subsequently, ten thousand cells were collected with FACS-Caliber and were analyzed using CELL QUEST_ 3.1 software (Becton Dickinson, USA). Appropriate negative controls were conducted to eliminate the possible effects of autofluorescence and nonspecific binding by the secondary antibody.

### Gene expression analysis in fibroblasts

RNeasy Mini Kit (Qiagen, Germany) was used for RNA isolation and quantitative real-time polymerase chain reaction (qRT-PCR) in cells treated with 0.5, 1 and 2% DMSO or considered as control. Extracted RNA from various groups was treated with DNase I (Fermentas, Germany) to remove any contaminating genomic DNA. Synthesis of cDNA was carried out according to previous recommendation (22). Briefly, 1 µg of total RNA was used for cDNA synthesis using random hexamer primer and RevertAid ™H First Strand cDNA Synthesis Kit (Fermentas, Germany). Real-time PCR was carried out with SYBR green (TaKaRa, Japan) in a thermal Cycler Rotor-Gene 6000 (Corbett, Australia). For each reaction, PCR mixture contained 5 µl Rotor-Gene SYBR Green PCR Master Mix (TaKaRa, Japan), 12.5 ng cDNA and 1.5 pmol of each primer in a final volume of 10 µl. Analysis of gene expressions was carried out by the ΔΔCT method and the relative levels of expression were normalized to *GAPDH* gene expression level. Primer sequences, annealing temperature and product size are listed in Table 1. 

**Table 1:** Primers used for the quantitative real-time polymerase chain reaction (RT-PCR) experiments


Gene	Primer sequence (5ˊ-3ˊ)	T_m_(^o^C)	Accession number

*OCT4*	F: TAAGAAAGGAATTGGGAAC	50	NW_005784454.1
	R: AGAACAAAGTGATGAGTG		
*NANOG*	F: TGGACTGGTTGGCTCTTATC	62	NW_005785373.1
	R: GCTGAGTTGAAGGAGAAGG		
*SOX2*	F: CCAAGAGAACCCTAAGATG	54	N/A
	R: TGTGTACTTATCCTTCTTCA		
*CDX2*	F: CACTACAGTCGCTACATCAC	56	NW_005785289.1
	R: TTTCCTTTGCTCTACGGTTC		
*TEAD4*	F: AAGTGGAGACCGAGTATG	55	NW_005785334.1
	R: GCTTGTGGATGAAGTTGAT		
*DNMT1*	F: GAAGCAGAATAAGAATCGG	54	NW_005783607.1
	R: TTTGAAGAGTCGTCTGGAA		
*DNMT3A*	F: TGGTCCTGGGCGTTAG	57	NW_005784665.1
	R: CCTGCTTTATGGAGTTCG		
*DNMT3B*	F: CGTCATCGCCCAGTGTT	54	NW_005785131.1
	R: TCTTCTCCCTCGCCATCT		
*β-ACTIN*	F: CCATCGGCAATGAGCGGT	58	NW_005783599.1
	R: CGTGTTGGCGTAGAGGTC		
*GAPDH*	F: GTTCAACGGCACAGTCAAG	60	NW_005785176.1
	R: TACTCAGCACCAGCATCAC		


T_m_; Melting temperature.

### Recovery and *in vitro* maturation of bovine oocytes

Bovine cumulus oocyte complexes (COCs) were recovered from slaughterhouse ovaries with 2-8 mm through 18 gauge needle attached with vacuum pump inside HEPES-buffered tissue culture medium 199 (H-TCM199, Sigma, USA) supplemented with 10% FBS. COCs with homogenous cytoplasm and with multiple layer of cumulus cells were selected for maturation, and incubated for 20 hours in TCM199 supplemented with 10% FBS, 2.5 mM sodium pyruvate (Sigma, USA), 10 µg/ml luteinizing hormone (LH, Sigma, USA), 10 µg/ml follicle-stimulating hormone (FSH, Sigma, USA), 1 µg/ml estradiol-17ß, 0.1 mM cysteamine, 100 ng/ml epidermal growth factor (EGF, Sigma, USA) and 100 ng/ml insulin- like growth factor (IGF, R&D, USA) at 38.5°C, 6% CO_2_, and maximum humidity.

### Interspecies somatic cell nuclear transfer procedure 

Procedure of iSCNT was carried out using manual oocyte enucleation using a pulled Pasteur pipette. In brief, matured oocytes were denuded by vortexing inside H-TCM199 supplemented with 300 IU/ml hyaluronidase for 3 minutes. For removing zona pellucida, denuded oocytes were exposed to 5 mg/ml pronase for 45 seconds followed by deactivated with H-TCM199+20% FBS for 20 minutes. The method of manual oocyte enucleation was used as described previously (23). Briefly, zona free oocytes were incubated in TCM199 supplemented with 4 µg/ml demecolcine for 1 hour in 38.5°C. Then, cytoplasmicprotrusion containing MII spindle, was removed byhand-held manual oocyte enucleation pipette. For nuclear transfer, nucleus-free bovine oocytes that have been successfully enucleated were transferred to dishes containing a droplets of H-TCM199 supplemented with 10 mg/ml phytohemagglutinin, and a well-rounded buffalo fibroblast cells were attached to membrane of enucleated oocytes. Subsequently couplets in fusion buffer free of Ca^2+^ and Mg^2+^ (290mOsm) were electrofused using sinusoidal electriccurrent (7 V/cm) for 10 sec followed by two directcurrents (1.75 kV/cm for 30 µ seconds and 1 seconddelay). After 30 minutes, oocyte activation inducedby incubation of reconstructed oocytes with 5 µMca-ionophore for 5 minutes followed by 4 hours incubation with 2 mM 6-dimethylaminopurine (6DAMP). Subsequently, activated reconstructed oocytes were cultured primarily in modified synthetic oviductal fluid (mSOF) for 12 hours (24). Thereafter, reconstructed oocytes (in a group of six) were culturedinside well containing 20 µ1 mSOF under mineraloil without epi-drugs at 38.5°C, 5% CO_2_, 5% O_2_ and humidified air for 6.5 days.

### Semi-quantitative assessment of DNA methylation in reconstructed embryos

Reconstructed oocytes (16 hours after activation) were washed in PBS-containing 0.1 mg/ml polyvinyl alcohol (PBS-PVA) and fixed for 20 minutes in 4% paraformaldehyde (Sigma, USA). Then permeabilization occurred with 1% Triton X-100 in PBS-PVA for 20 minutes at RT. For incorporation of 5-methylcytidine antibody into DNA, reconstructed oocytes were treated with 4 N HCl for 30 minutes at RT and then neutralized for 20 minutes with Tris-HCl buffer (100 mM in pH=8.0). For blocking non-specific binding sites, reconstructed oocytes were incubated in blocking solution [PBS-PVA containing 1% BSA (Sigma, USA) and 10% goat serum] for 2 hours at RT. Incubation of reconstructed oocytes with primary and secondary antibodies was conducted according to the protocol explained earlier. Finally, reconstructed oocytes were exposed to Hoechst and pixel intensity of pseudo-pronucleus was evaluated using Image J. software [National Institute of Mental Health, Bethesda, Maryland, USA] (25). Appropriate controls were included to check the autofluorescence of the first and second antibodies. 

### Gene expression analysis in interspecies somatic cell nuclear transfer blastocysts 

RNeasy Micro Kit was used for RNA extraction from blastocyst embryos as described previously (26) (Qiagen, Germany). Reverse transcription was immediately performed using a QµantiTect Reverse Transcription (RT) Kit (Qiagen, Germany). The cDNA was stored at -70°C and analysed by quantitative RTPCR (qRT-PCR) using standard conditions. Relative expression was calculated using Ct values which were normalized against ß-actin (reference gene). Three replicates were done for each PCR reactions. ΔΔCT method was used to estimate fold changes between genes of target following RT-qPCR. The value comparative threshold cycle (CT) denotes the threshold cycle, and .CT was calculated as CT of the target gene -CT of reference gene. Fold change in gene expression was calculated using 2^-ΔΔCT^, where ΔΔCT was calculated as ΔCT. Primer sequences, annealing temperature and product size are listed in Table 1. 

### Experimental design 

A non-toxic and non-effective concentration of DMSO (0.5, 1, 2 and 4%) for treatment of buffalo- bovine reconstructed oocytes, were determined using the tests for cell viability and intensity of methylation as well as the expression levels of DNMTs family on fibroblast cells. Next, the effects of exposing reconstructed oocytes, for 16 hours after activation, to DMSO (0.5%) on the respective level of 5-methylcytosine, cleavage rates and blastocyst rates and gene expression (pluripotency genes: *OCT4, NANOG, SOX2,* and trophectodermal genes: *CDX2* and *TEAD4*) of produced blastocysts were investigated.

### Statistical analysis 

The response variables had a discrete nature with a binomial distribution; therefore, all percentage data were subjected to ArcSin transformation. Cell viability, epigenetic level of treated fibroblasts were analyzed using one-way ANOVA followed by Tukey multiple comparison post hoc test in SPSS (SPSS, Version 20, IBM, USA). Epigenetic level of reconstructed oocytes and gene expression in fibroblast cells and blastocyst and developmental rates of experimental groups were compared using independent samples t test. Data were presented as mean ± SEM. P<0.05 were considered as statistically significant.

## Results

### Cell viability 

**Fig.1:**
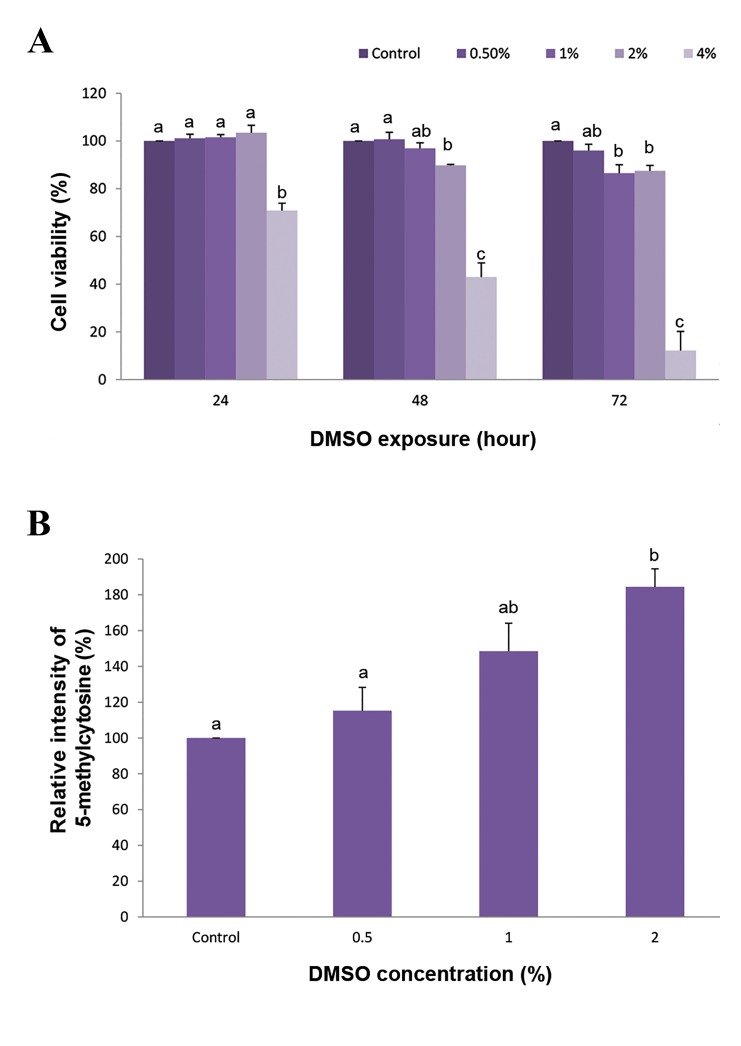
Effect of different concentrations of DMSO on buffalo fibroblast cells. A. Cell viability of fibroblast buffalo cells exposed to different concentrations of DMSO for 24, 48 and 72 hours and B. Relative intensity of 5-methylcytosine in buffalo fibroblast cells following exposure to various concentrations of DMSO for 24 hours. ^a, b^; Different letters indicates significant differences (P<0.05) and DMSO; Dimethyl sulfoxide.

The possible toxicity effect of DMSO on the viability of buffalo fibroblast cells, was determined using MTS assay following exposure of buffalo fibroblast cells to 0, 0.5, 1, 2 and 4% DMSO for 24, 48 and 72 hours. Exposure of fibroblasts to 0.5% DMSO for 24, 48 and 72 hours did not reveal any adverse effect on the cell viability. However, cell viability started to decline following exposure to 1 (86.51 ± 3.57%) after 72 hours, 2 (89.80 ± 2.71%) after 48 hours and 4% (70.86 ± 3.17%) DMSO after 24 hours compared to control (Fig .1A, P<0.05).

### DNA methylation in buffalo fibroblasts

To investigate the possible epigenetic effect of DMSO on global DNA methylation, buffalo fibroblast cells were treated for 24 hours with nontoxic doses of DMSO (0.5, 1 and 2%), according to the cytotoxicity results elaborated in the cell viability experiment of the present study. The relative intensity of 5-methylcytosine increased in a dose dependent manner after treating buffalo fibroblast cells with DMSO. The level of 5-methylcytosine in 0.5 and 1% DMSO (115.24 ± 13.05 and 148.46 ± 15.68% respectively, Fig .1B) was not significantly higher than control group (P>0.05). However, this increase reached a significant level after treating the fibroblast cells with 2% DMSO (184.46 ± 10.07%, P<0.05, Fig .1B). 

### Gene expression of DNA methyl-transferase family in buffalo fibroblasts 

**Fig.2:**
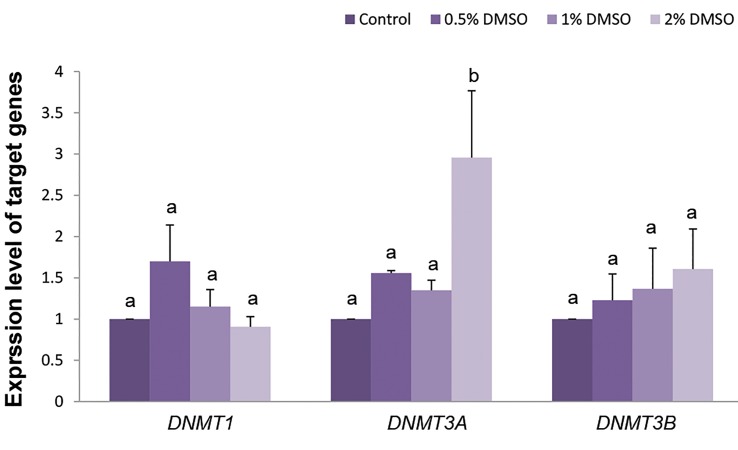
Real-time reverse-transcriptase polymerase chain reaction (PCR) gene expression analysis in buffalo fibroblast cells treated with various concentrations of DMSO for 24 hours. DMSO; Dimethyl sulfoxide and ^a, b^; Different letters indicates significant differences (P<0.05).

In order to understand the reason of elevated level of 5-methylcytosine in 1 and 2% DMSO treated cells, we designed an experiment to investigate the effect of nontoxic doses of DMSO (0.5, 1, and 2%) on the expression of DNMTs family (*DNMT1, DNMT3A* and *DNMT3B*). Relative mRNA expression of *DNMT1* and *DNMT3B* were similar in control and various concentrations of DMSO (Fig .2, P>0.05). However, mRNA expression of *DNMT3A* was greater in 2% DMSO treated cells compared to other groups (Fig .2, P<0.05). 

### *In vitro* development of buffalo-bovine interspecies somatic cell nuclear transfer 

In order to investigate the possible effect of DMSO (0.5%, the safe concentration of DMSO on buffalo fibroblast cells achieved in the previous experiment of the present study) on cleavage and blastocyst rates of buffalo-bovine iSCNTembryos, reconstructed oocytes were treated with 0.5% DMSO for 16 hours after activation. There was no difference between experimental groups in cleavage (control: 87.2 ± 1.59% and treatment: 86.9 ± 1.34%) and blastocyst rates(control: 4.8 ± 0.91% and treatment: 4.6 ± 0.74%, P>0.05, Table 2). 

### DNA methylation in buffalo-bovine reconstructed oocytes 

**Fig.3:**
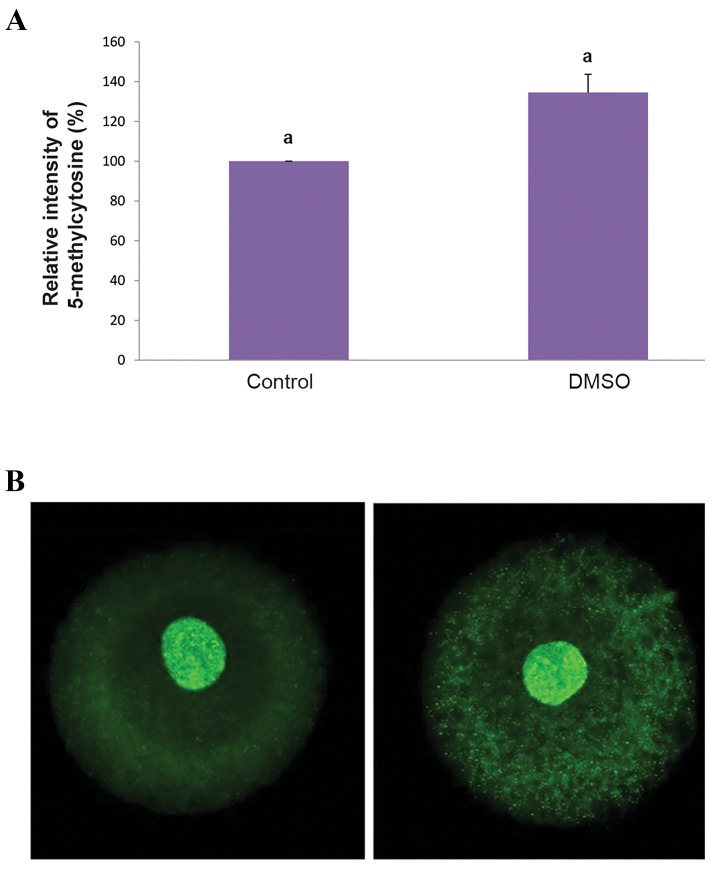
Semi-quantitative analysis of fluorescence intensity of 5-methycytosine in buffalo-bovine reconstructed oocytes. A. Relative intensity of 5-methycytosine in buffalo-bovine reconstructed oocytes after exposure to 0.5% DMSO for 16 hours post activation in compare to control, B. Immunofluorescence images of 5-methylcytosine in buffalo- bovine reconstructed oocytes exposed to 0.5% DMSO for 16 hours post activation in compare to and B'. Control (scale bar: 50 µm). DMSO; Dimethyl sulfoxide. Similar letters indicates non-significant differences.

The exposure of buffalo-bovine reconstructed oocytes toDMSO (0.5%) for 16 hours post activation did not affect DNAmethylation, assessed by the intensity of 5-methylcytosinein pseudo-pronucleus of 1-cell iSCNT embryos (134.55 ± 9.15%) compared to control (Fig .3, P>0.05).

### Expression of developmental genes in blastocysts

**Fig.4:**
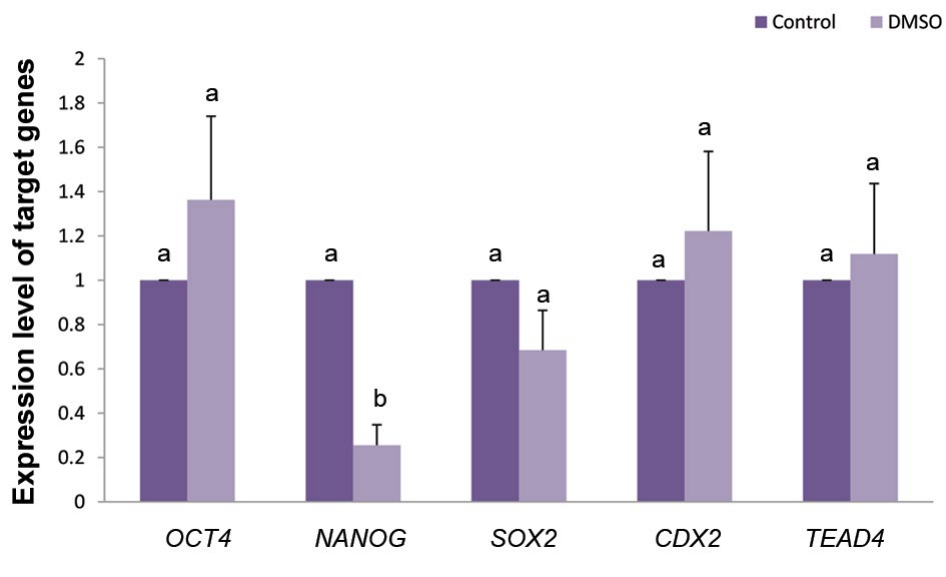
Real-time reverse-transcriptase polymerase chain reaction (PCR) gene expression analysis in blastocysts derived from DMSO (0.05%) compared to control. DMSO; Dimethyl sulfoxide and ^a, b^; Different letters indicates significant differences (P<0.05).

In order to evaluate the quality of derived iSCNT blastocystafter exposure of reconstructed oocytes to 0.5% DMSO, themRNA expression of pluripotent genes (*OCT4, SOX2* and *NANOG*) and trophectodermal genes (*CDX2* and *TEAD4*) were assessed in both control and treated groups. The relative expression of *OCT4, SOX2, CDX2* and *TEAD4* genes inblastocyst stage was not different between DMSO and controlgroups (Fig .4, P>0.05). However, expression of *NANOG* was significantly lower in DMSO treated group compared tocontrol (Fig .4, P<0.05). 

**Table 2:** Development of buffalo-bovine iSCNT embryos after exposing reconstructed oocytes to 0.5 % DMSO


Group	Reconstructed oocytes	Cleaved oocytes	Blastocyst

Control-iSCNT	583	456 (87.2 ± 1.59)^a^	22 (4.8 ± 0.91)^a^
DMSO-iSCNT	679	525 (86.9 ± 1.34)^a^	24 (4.6 ± 0.74)^a^


Values with the same superscripts within column did not have significant differences (P>0.05). iSCNT; Interspecies somatic cell nuclear transfer and DMSO; Dimethyl sulfoxide. Data were presented as number (% ± SEM).

## Discussion

The main objective of the present study was to examine the effect of DMSO on epigenetic status of treated somatic cells, buffalo-bovine iSCNT reconstructed oocytes as well as the cleavage and blastocyst rates of these reconstructed oocytes. Initial attempts to achieve such objectives was to elaborate the safest dose of DMSO for treating the reconstructed oocytes. Supplementation of culture medium with DMSO could have substantial adverse effect on the cell viability depending on the amount and exposure time. Accordingly, significant decrease in cell viability was noticed following exposure of fibroblast cells to 2 and 4% DMSO. This is in agreement with previous studies in which DMSO had toxic effect at these concentrations (27, 28). However, cell viability was not affected by 0.5% DMSO concentration. This is consistent with the report investigated in rat (28). 

In the present study, DNA methylation in fibroblast treated cells amplified by increasing the concentration of DMSO. The highest level of DNA methylation was observed at 2% concentration of DMSO, which was associated with a significant increase in the expression of *DNMT3A*. However, the lower concentration of DMSO (0.5%) did not affect the methylation nor the gene expression of *DNMTs* family. Consistent with our results, Iwatani and colleagues (10) demonstrated the upregulation of mRNA and protein of *DNMT3A* by DMSO in embryonic bodies derived from embryonic stem cells. Furthermore, they showed that "DMSO affected DNAmethylation status at multiple loci, inducing hypomethylation as well as hypermethylation using restriction landmark genomic scanning" (10). Moreover, Yokochi and Robertson have shown that DMSO could increase the activity of *DNMT3A* and *DNMT1* enzymes in *in vitro* condition (19). This is in agreement with the result of the present study when 2% DMSO increased the activity of *DNMT3A*. Thaler’s report (12) showed that DMSO increased global and gene-specific DNA hydroxymethylation levels and expression of TET and GADD45A genes in pre-osteoblastic MC3T3-E1 cells. In addition, their results revealed a loss of 5-methylcytosine on Fas (pro-apoptotic gene) and *Dlx5* (early osteoblastic factor) promoters as well as an increase in 5-hmC. 

In the current study, there was a slight, but not significant, increase in level of DNA methylation in treated buffalo- bovine reconstructed oocytes (0.5% DMSO) compared to control group. However, in the embryonic bodies of mice, any concentrations of DMSO, between 0.02 and 1%, could alter the level of methylation significantly (10). 

There was no adverse effect of 0.5% DMSO on cleavage and blastocyst rates. This confirms that the safe concentration of DMSO was selected throughout the dose-response study conducted on buffalo fibroblast cells. Interestingly, Wakayama has shown that "addition of 1% DMSO to the activation medium during SCNT procedure significantly improved the frequency of development to the blastocyst stage and full term" (9). 

The effect of DMSO (0.5%) on mRNA expression of some developmentally important genes (*OCT4, NANOG, SOX2, CDX2* and *TEAD4*) in buffalo-bovine iSCNT blastocysts was assessed using real time RT-PCR. The expression of *NANOG* decreased in DMSO treated reconstructed oocytes compared to control. This reduction in expression of *NANOG* in reconstructed oocytes may be related to the slight global hyper-methylation of genome. The level of methylation is very important throughout embryonic development. In mice, before implantation the embryos undergoes a wave of DNA demethylation, which erases the inherited parental methylation pattern, while after implantation the embryos undergo a wave of de novo DNA methylation that establishes a new DNA methylation pattern (29, 30). In the present study the slight global hyper-methylation in reconstructed oocytes may be related to the expression of *DNMT3A* (based on the effect of 2% DMSO on buffalo fibroblast). The expression of *DNMT3A* significantly expressed after day 10 in mouse embryo (31), but not for the *DNMT3B*, and any error in the expression of these genes could affect the fate of embryonic development (32).

While expression of *OCT4* is highly regulated by the methylation status of its promoter, the mRNA expression of this gene in the present study remained unchanged in DMSO group compared to control. In this notion, Iwatani and colleagues have shown that thousands of loci remained unchanged in EBs after treatment with DMSO (10), which can explain the unchanged expression of *OCT4* in DMSO group compared to control. 

## Conclusion

The results of this study revealed the epigenotoxic effect of DMSO in buffalo fibroblast cells and reconstructed oocytes derived from buffalo-bovine iSCNT procedure. DMSO at the concentration of 2% could induce a global DNA hyper-methylation, possibly through high expression of *DNMT3A* in treated fibroblast cells. However, there was slight global DNA hyper-methylation in reconstructed oocytes after treatment with 0.5% DMSO. This phenomenon may account for lower expression of *NANOG* in iSCNT derived blastocysts. Collectively, these results may have some implications and precaution for using DMSO as a solvent or cryoprotectant in biomedical sciences. 
